# Voxel-Wise Analysis of Structural and Functional MRI for Lateralization of Handedness in College Students

**DOI:** 10.3389/fnhum.2021.687965

**Published:** 2021-08-13

**Authors:** Haha Wang, Hong Zhou, Yihao Guo, Lei Gao, Haibo Xu

**Affiliations:** ^1^Department of Radiology, Zhongnan Hospital of Wuhan University, Wuhan, China; ^2^Siemens MR Collaboration, Siemens Healthcare, Guangzhou, China

**Keywords:** handedness, voxel-mirrored homotopic connectivity, asymmetry index, magnetic resonance imaging, lateralization

## Abstract

The brain structural and functional basis of lateralization in handedness is largely unclear. This study aimed to explore this issue by using voxel-mirrored homotopic connectivity (VMHC) measured by resting-state functional MRI (R-fMRI) and gray matter asymmetry index (AI) by high-resolution anatomical images. A total of 50 healthy subjects were included, among them were 13 left-handers, 24 right-handers, and 13 mixed-handers. Structural and R-fMRI data of all subjects were collected. There were significant differences in VMHC among the three groups in lateral temporal-occipital, orbitofrontal, and primary hand motor regions. Meanwhile, there were significant differences in AI that existed in medial prefrontal, superior frontal, and superior temporal regions. Besides, the correlation analysis showed that the closer the handedness score to the extreme of the left-handedness (LH), the stronger the interhemispheric functional connectivity, as well as more leftward gray matter. In general, left/mixed-handedness (MH) showed stronger functional homotopy in the transmodal association regions that depend on the integrity of the corpus callosum, but more variable in primary sensorimotor cortices. Furthermore, the group differences in VMHC largely align with that in AI. We located the specific regions for LH/MH from the perspective of structural specification and functional integration, suggesting the plasticity of hand movement and different patterns of emotional processing.

## Introduction

Human brain behaviors exhibit significant lateralization (Ocklenburg et al., 2014), including language (Ocklenburg et al., 2013; Yazbek et al., 2020), visuospatial (Vogel et al., 2003; Tokgoz et al., 2020), memory (Babiloni et al., 2006; Zhou et al., 2020), attention (Duecker et al., 2013), and emotional processing (Lindell, 2013). Anatomically, the brain basis of these lateralized behaviors is supposed to be associated with prominent structural asymmetries, including the lateral fissure (Rubens et al., 1976; Toga and Thompson, 2003; Essen, 2005) and the supratemporal sulcus (Shapleske et al., 1999; Hirayasu et al., 2000); however, the structure and the function of lateralization behavior, such as the handedness, is still an open question.

Handedness is an important feature of lateralization in humans. More than 90% of the population is right-handed. The motor asymmetry of the hand appears in prenatal development and has been linked to genetic origin (Corballis, 1997) and brain size and social network expansion (Halpern et al., 2005). Furthermore, previous research (Willems et al., 2014) suggests that left-handers tend to weaken brain lateralization, as compared to right-handers. The evidence in these documents suggests that left-handers may have distinct interhemispheric interaction patterns and macroscale organization, both structurally and functionally.

Functionally, interhemispheric interaction is critical to efficient communication and cooperation for human behaviors and cognition (Li et al., 2017; Qiu et al., 2017a,b; Gao et al., 2019; Ye et al., 2020). Structurally, brain lateralization is an important basis for functional specification and interhemispheric interaction (Postema et al., 2019; Dutta et al., 2020; Güntürkün et al., 2020; Jber et al., 2021). Resting-state functional magnetic resonance imaging (R-fMRI) is a powerful tool for non-invasively studying such neuroscience questions. Among the R-fMRI analysis methods, voxel-mirrored homotopic connectivity (VMHC) (Zuo et al., 2010), which reflects the homotopic functional connectivity between the two hemispheres by calculating the correlation coefficients of voxels in symmetrical positions, has been widely used in nervous system diseases in recent years (Hoptman et al., 2012; Hua-Jun et al., 2014; Wei et al., 2014; Xu et al., 2014; Chen et al., 2015; Hu et al., 2015; Jiabao et al., 2019; Yun et al., 2019; Zhao et al., 2020; Wu et al., 2021). Furthermore, voxel-based morphometry (VBM) using high-dimensional registration can also perform hemispheric asymmetry analysis and generate gray matter asymmetry index (AI) at the voxel level (Florian et al., 2015). Both the VMHC and the gray matter AI show good test–retest reliability in discovering plasticity and disease-related changes (Zuo et al., 2019). In this study, we explored how and to what extent the handedness is coupled with interhemispheric functional integration and hemispheric gray matter lateralization across the full range of handedness scores (i.e., from the left extreme −100 to the right extreme 100). Considering the prominent hand movement coordination and higher mental illness incidence (Goez and Zelnik, 2008; Postema et al., 2019; Dutta et al., 2020; Jber et al., 2021), we, therefore, hypothesized that the extent of left-handedness tightly aligns with homotopic connectivity and gray matter lateralization in brain regions involving in hand sensorimotor, visual motion, and emotional processing.

## Materials and Methods

### Subjects

Fifty volunteers aged between 18 and 32 years participated in the study through campus advertisement, including 13 left-handedness (LH), 24 right-handedness (RH), and 13 mixed-handedness (MH). All subjects underwent Mini-Mental State Exam (MMSE) (Tombaugh and McIntyre, 1992) and handedness assessment. The handedness was assessed using a modified Chinese version of the Handedness questionnaire, and hand preference was assessed for 10 different activities (Li, 1983). The inclusion criteria were the following: (1) the MMSE score > 24; (2) no history of psychiatric or neurological illness; (3) satisfied the conditions of MRI examination and signed informed consent. The details of the three groups are presented in Table 1. The exclusion criteria were the following: (1) the MMSE score ≤ 24; (2) history of psychiatric or neurological illness; (3) any contraindications for MR scan; (4) low education level (<6 years).

**Table 1:** Summary of the demographic and questionnaire score.

	**Left (*n* = 13)**	**Mixed (*n* = 13)**	**Right (*n* = 24)**	**Group Comparisons**		***Post hoc***
	**Mean ± SD**	**Mean ± SD**	**Mean ± SD**	**Statistic**	***p***	
Gender (male/female)	8/5	4/9	11/13	χ^2^ = 2.48	0.290	_
Age (years)	24.46 ± 2.70	22.69 ± 3.34	24.83 ± 3.30	*F* = 2.24	0.117	_
Handedness score	−71.23 ± 15.83	−7.96 ± 23.75	99.58 ± 2.04	*F* = 636.90	<0.001	Right>Mixed>Left
MMSE score	30	30	30	*F* = 0	>0.99	_

This study was approved by the local Medical Ethics Committee in Zhongnan Hospital of Wuhan University and informed written consent was signed by all participants.

### Handedness Questionnaire

Referring to the Annett hand preference questionnaire (Annett, 1970) and the Oldfield Edinburgh handedness inventory (Oldfield, 1971), a Chinese scholar (Li, 1983) has developed a handedness questionnaire for Chinese people, which contains a series of daily hand tasks. There are a total of ten items, each with two points. If you usually use the left (right) hand, the left (right) hand will get two points. If the two hands are used frequently, then the left and right hands will each get one point, and finally count the total score of the left and right hands. The total score of the right hand minus the total score of the left hand is the difference score. The difference score is then divided by the total score (the sum of the left and right score) and multiplied by 100 to generate the final handedness score. Subjects scoring < −40 are LH, from −40 to 40 are MH, and higher than 40 are RH.

### MRI Data Acquisition

MRI data were collected using a 3T MRI scanner (MAGNETOM Trio, Siemens Healthcare, Erlangen, Germany), equipped with a 32-channel head coil. All subjects were asked to lie down in the supine position, with eyes opened and relaxed. The R-fMRI data were acquired using an echo-planar imaging (EPI) sequence consisting of 240 volumes in 8 min, 32 axial slices with a thickness of 3.5 mm, no slice gap, repetition time (TR) = 2000 ms, echo time (TE) = 30 ms, flip angle (FA) = 90°, field of view (FOV) = 196 × 196 mm^2^, and matrix size = 64 × 64. Meanwhile, three-dimensional (3D) high-resolution T1-weighted images were acquired using magnetization-prepared rapid acquisition gradient echo (MPRAGE) sequence for spatial normalization and AI calculation, with TR = 2250 ms, TE = 2.26 ms, FA = 9°, FOV = 256 × 256 × 176 mm^3^, and matrix size = 256 × 256 × 176.

### Resting-State-Functional MRI Data Preprocessing

The acquired data were preprocessed using Data Processing Assistant for R-fMRI (DPARSF, version 3.2; http://restfmri.net/forum/DPARSF) (Yan and Zang, 2010) and Statistical Parametric Mapping (SPM version8; http://www.fil.ion.ucl.ac.uk/spm) on MATLAB (MathWorks Inc., Natick, MA, USA) platform, which consisted the following: (1) NIFTI format conversion; (2) the removal of first 10 volumes; (3) slice timing; (4) head-motion correction with a 24-parameter linear and non-linear transformations (none of subjects with the data of each side moving to the head > 1 mm and rotation > 1° was excluded during the MRI scan); (5) T1 structural images were coregistrated to functional images and segmented into white matter, gray matter, and cerebrospinal fluid; (6) nuisance covariates regression (including the 24-parameter head motion model, white matter, cerebrospinal fluid); (7) spatial normalization to Montreal Neurology Institute (MNI) space and resampling to 3 × 3 × 3 mm^3^; (8) smooth with the Gaussian kernel of 6 mm; (9) filtering (0.01–0.08 Hz).

### T1 Data Preprocessing

After visual inspection of the quality of high-resolution T1-weighted anatomical images of all subjects, the images were manually reorientated and aligned with the anterior commissure-posterior commissure (AC-PC) line. Then, we used the method provided by Kurth et al. (Florian et al., 2015) using VBM (VBM version8; http://dbm.neuro.uni-jena.de/vbm.html) and SPM8 software, including the following steps: (1) NIFTI format transformation; (2) segment for gray matter and white matter; (3) left-right flip of gray matter and white matter; (4) creation of a symmetric Diffeomorphic Anatomic Registration Through Exponentiated Lie algebra algorithm (DARTEL) template (Ashburner, 2007) by using the flipped images and the original images; (5) registration of the original and flipped images into the symmetric DARTEL template; (6) calculation of the total intracranial volume (TIV).

### VHMC Calculation

The images were normalized to a symmetric template before VMHC analysis. First, all the normalized images were averaged to obtain an average T1 template, then, the T1 template was used to average the left and right hemispheres to obtain a symmetric template. Finally, all the preprocessed functional images were registered to the obtained symmetric template (Zuo et al., 2010). The time series of each voxel in the hemisphere was extracted and the correlation coefficient of the voxels in the symmetric position on the left and right sides was calculated, then converted into zVMHC maps by Fisher Z transformation for further statistical analysis.

### AI Calculation

The gray matter AI images were calculated within the right hemispheric mask, with the following formula:

AI = (right-left)/0.5 × (right + left),

where AI > 0 indicates that the gray matter volume of the right hemisphere is larger than that of the left hemisphere, and the gray matter is right-lateralized. Similarly, AI <0 indicates that the images are left-lateralized. The abovementioned AI images were smoothed with a full width at half maximum (FWHM) of 8 mm Gaussian kernel for subsequent statistical analysis.

### Statistical Analysis

Statistical Package for the social Sciences (SPSS) (https://www.ibm.com/products/spss-statistics) was used for statistical analysis of demographic statistics and handedness scores. To determine any possible differences in VMHC between the three groups, we first performed a one-way analysis of covariance (ANCOVA) with SPM8, gender and age as covariates, limiting them to the right hemisphere. Then, to determine the directions of between-group differences, we extracted the VMHC values of the significant clusters and performed *post-hoc* analyses. For AI images, ANCOVA was conducted with SPM8. Gender, age, and TIV were controlled and confined to the right hemisphere. After that, to determine the directivity of these differences between groups, we extracted the AI values of the above significant clusters and performed *post-hoc* analysis to determine the differences between any two groups. We finally examined the whole-brain structural and functional correlates of handedness scores. With the handedness score as a function of imaging measures, we included the handedness scores of all subjects (from −100 to +100 as a spectrum distribution of handedness) and calculated whole-brain voxel-level Pearson's correlations between handedness scores and VMHC or AI. Statistical analysis was performed with a voxel threshold of *p* < 0.005, and multiple comparisons were corrected based on a family-wise error (FWE) with a cluster threshold of *p* < 0.050.

## Results

### Demographic Statistics

Table 1 shows the demographic and handedness behavioral data.

### Between-Group Differences in VHMC

**Figure 1:**
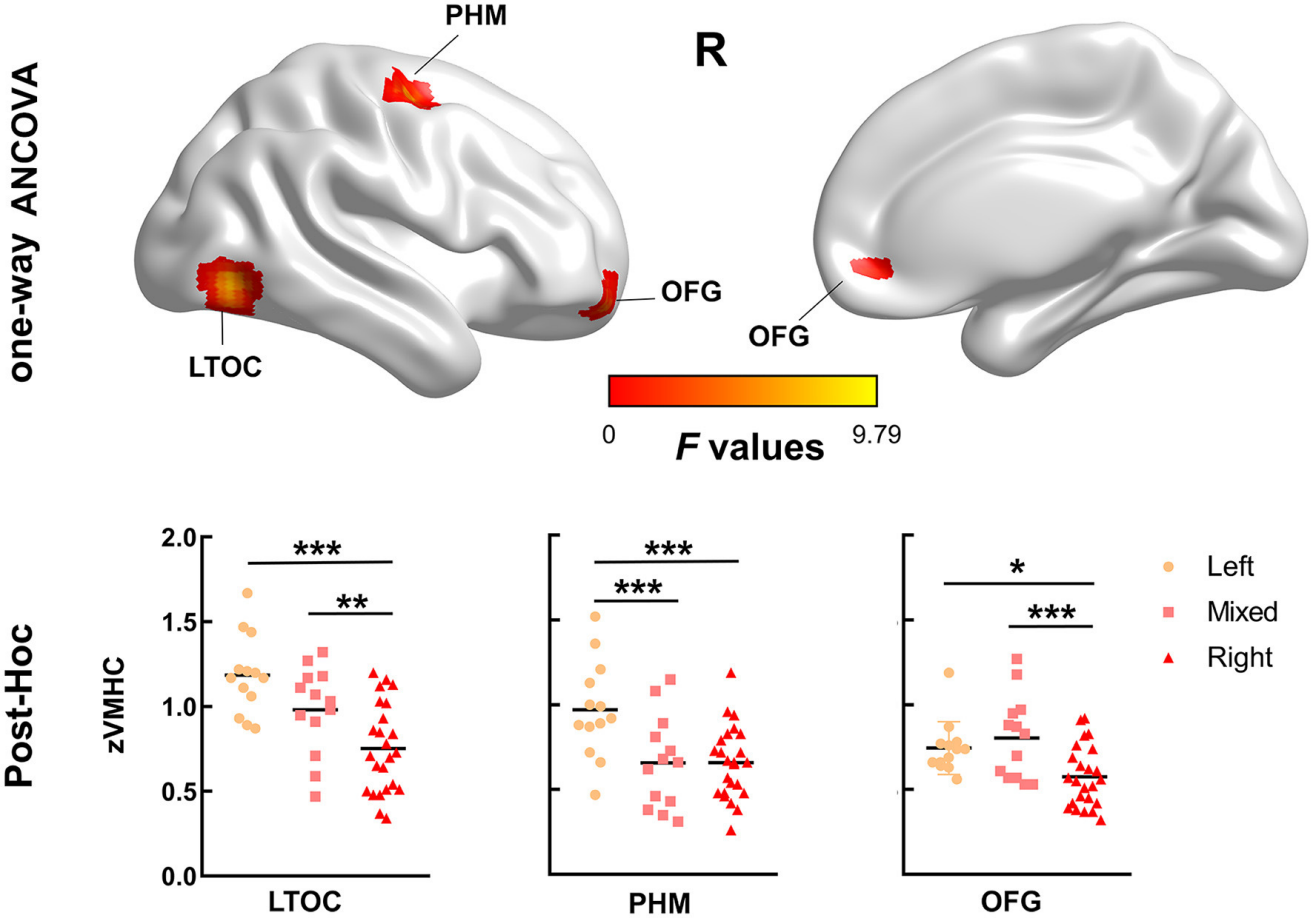
Brain areas with significant differences in voxel-mirrored homotopic connectivity (VMHC) values among the three groups. There were significant differences in VMHC among the three groups in lateral temporal-occipital cortices (LTOC), primary hand motor region (PHM), and orbitofrontal cortex (OFC) (family-wise error, FWE cluster corrected, *p* < 0.050). We extracted the VMHC values of the above significant clusters and performed *post-hoc* analysis to determine the between-group differences. Specifically, both left-handedness (LH) and mixed-handedness (MH) showed enhanced interhemispheric functional connectivity in LTOC and OFC compared with right-handedness, and the interhemispheric functional connectivity of LH in the PHM region was enhanced than that of mixed/right-handedness. (*: *p* < 0.050, **: *p* < 0.010, ***: *p* < 0.001).

Between-group differences in VMHC are shown in Figure 1 and Table 2. There were significant differences in VMHC among the three groups in lateral temporal-occipital cortices, orbitofrontal cortex, and primary hand motor region (including precentral gyrus, middle frontal gyrus, and superior frontal gyrus). Specifically, both LH and MH showed greater homotopic connectivity in lateral temporal-occipital cortices and orbitofrontal cortex than that of the RH, and the LH showed greater homotopic connectivity in the primary hand motor region than that of the MH/RH.

**Table 2:** Voxel-mirrored homotopic connectivity (VHMC) statistics for the groups.

**Regions**	**MNI coordinates**			**Cluster size**	***F-value***	**Left (*n* = 13)**	**Mixed (*n* = 13)**	**Right (*n* = 24)**
	**X**	**Y**	**Z**			**Mean ± SD**	**Mean ± SD**	**Mean ± SD**
LTOC	42	−72	−3	37	9.37	1.17 ± 0.07	1.00 ± 0.07	0.76 ± 0.05
OFC	12	48	−9	23	8.06	0.94 ± 0.06	0.67 ± 0.06	0.66 ± 0.05
PHM	33	−6	60	41	9.80	0.71 ± 0.05	0.85 ± 0.05	0.57 ± 0.04

*The coordinates were shown as stereotaxic coordinates referring to the space of the Montreal Neurological Institute (MNI)*.

*LTOC: lateral temporal-occipital cortices; OFC: orbitofrontal cortex; PHM: primary hand motor*.

### Between-Group Differences in Gray Matter Asymmetry Index

**Figure 2:**
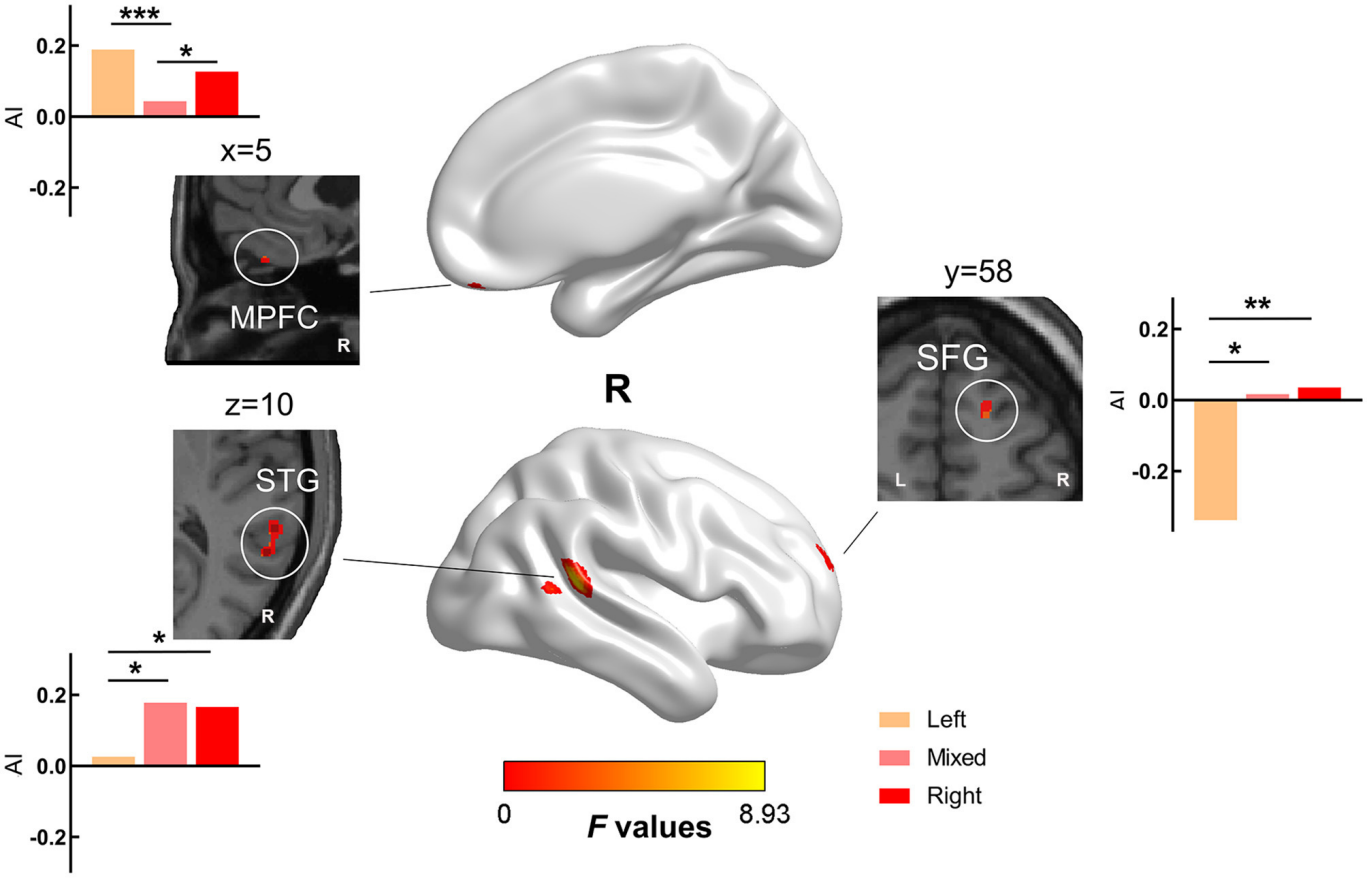
Brain areas with significant differences in AI values among the three groups. There were significant differences in gray matter lateralization in the medial prefrontal cortex (MPFC), superior frontal gyrus (SFG), and superior temporal gyrus (STG) among the three groups. (FWE cluster corrected, *p* < 0.050). We extracted the AI values of the above significant clusters and performed *post-hoc* analysis to determine the differences between the two groups. We found that LH and RH showed a rightward shift of gray matter compared with MH in MPFC, and LH showed a leftward shift in SFG and STG compared with mixed/RH. (*: *p* < 0.050, **: *p* < 0.010, ***: *p* < 0.001).

The one-way ANCOVA results of the gray matter AI are shown in Figure 2 and Table 3. The ANCOVA analyses identified significant clusters with altered gray matter AI in the medial prefrontal, superior frontal, and superior temporal regions. Both the LH and RH showed rightward shift of gray matter in the medial prefrontal cortex compared with MH, and LH showed leftward shift in the superior frontal gyrus (SFG) and superior temporal gyrus (STG) compared with MH/RH.

**Table 3:** Asymmetry index (AI) statistics for the groups.

**Regions**	**MNI coordinates**			**Cluster size**	***F-value***	**Left (*n* = 13)**	**Mixed (*n* = 13)**	**Right (*n* = 24)**
	**X**	**Y**	**Z**			**Mean ± SD**	**Mean ± SD**	**Mean ± SD**
MPFC	2	50	−26	15	6.20	0.19 ± 0.13	0.04 ± 0.12	0.13 ± 0.08
SFG	17	57	20	31	7.51	−0.34 ± 0.61	0.02 ± 0.19	0.04 ± 0.17
STG	59	−41	12	165	8.93	0.02 ± 0.26	0.18 ± 0.17	0.17 ± 0.13

*The coordinates were shown as stereotaxic coordinates referring to the space of the Montreal Neurological Institute (MNI)*.

*MPFC: medial prefrontal cortex; SFG: superior frontal gyrus; STG: superior temporal gyrus*.

### Whole-Brain Voxel-Wise Correlation Analyses

**Figure 3:**
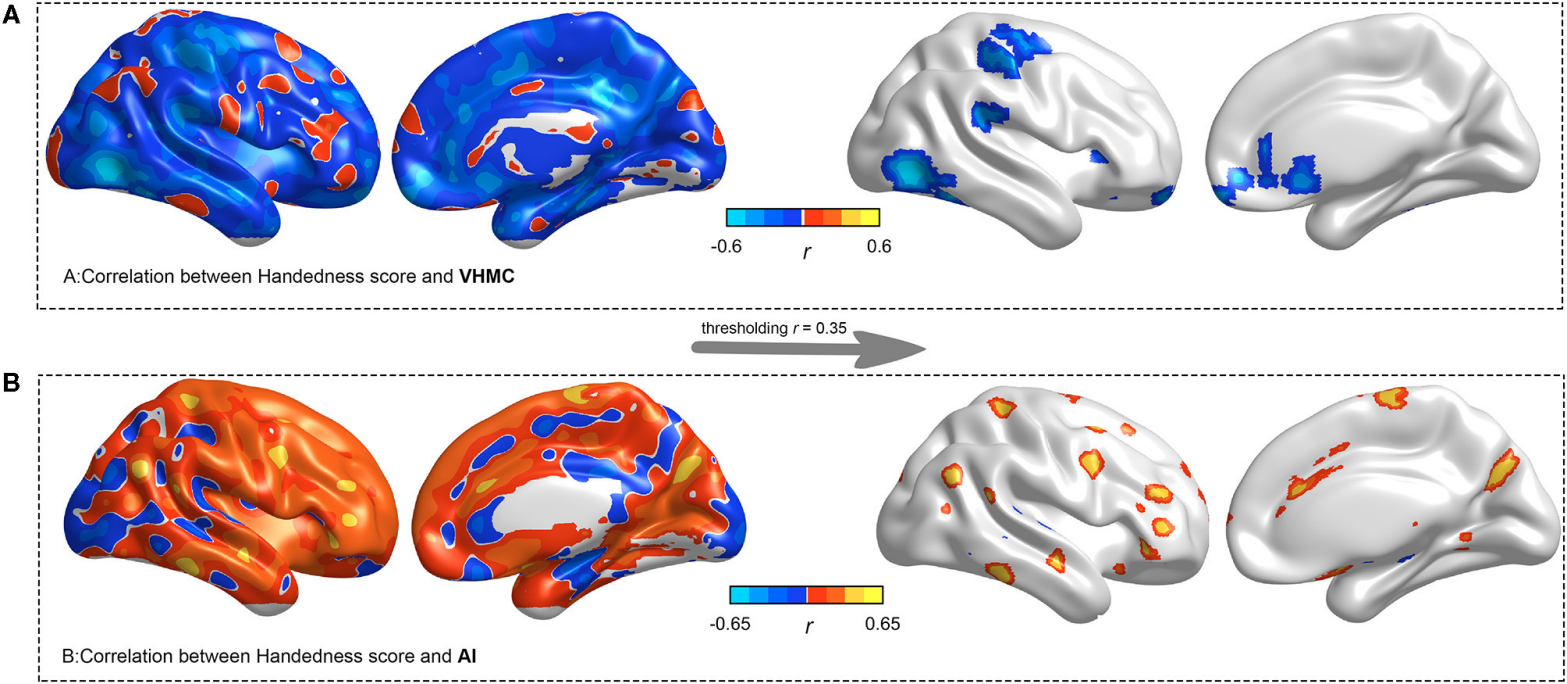
Voxel-wise whole-brain linear regression analysis between VMHC/AI and handedness scores. The statistical significance was voxel *p* < 0.005 and corrected at FWE cluster *p* < 0.050. The left panel indicates unthresholded voxel-wise correlation coefficients, and the right panel indicates thresholded (*r* = 0.35) voxel-wise correlation coefficients.

We found significantly negative correlations between handedness score and VMHC in the middle temporal, the posterior cingulate, the fusiform, the orbitofrontal, and the primary hand motor regions (Figure 3). Meanwhile, we found significantly positive correlations between handedness score and AI, widely distributed in the lateral temporal, the hand motor, the cingulate, and the angular regions. The whole-brain voxel-wise regression was voxel *p* < 0.005 and corrected at FWE cluster *p* < 0.050.

## Discussions

Using voxel-wise homotopic functional connectivity and gray matter volume asymmetry (lateralization), we examined the interhemispheric functional and structural correlates of handedness. We found a structure-function coupling into the hand motor, visual motion, and limbic regions toward LH and MH. These findings correlate the handedness gradient between interhemispheric functional integration and hemispheric structural specification, suggesting distinct brain organizational principles in LH and MH, especially in the regions subserving hand motor, higher visual, and emotional processing. The current findings are in line with this hypothesis and previous reports (Foundas et al., 1998; Hervé et al., 2006; Amunts, 2010). The involved areas were generally considered to support motor of hands, language, visual-auditory-tactile integration, and emotional processing.

In terms of interhemispheric functional connectivity, one main result was altered VMHC in a cluster comprising the hand motor area, and this brain cluster extended to the SFG and the posterior part of the middle frontal gyrus. This result is of great significance because this remarkable cluster is not only an important anatomical focus of hand movement but also contains the “graphic motor image center” in the middle frontal gyrus first described by Exner in 1881 (Franck-Emmanuel et al., 2010). The primary hand motor area is related to writing and handedness. Increased interhemispheric functional connectivity with LH in this area might be related to its daily left-handed behavior, which suggests that LH has stronger interhemispheric functional integration and neural signal coordination. This is consistent with the results of correlation analysis. In the correlation analysis, there were significant negative correlations in the primary hand motor region between handedness score and VMHC, suggesting that the closer the handedness score to the extreme of the LH, the stronger the connection between the left and the right hemispheres in the primary hand motor region.

We also found altered functional homotopy in lateral temporal-occipital junction. The lateral temporal-occipital junction, also termed as the MT area, is responsible for advanced visual processing including pattern, face, and visual motion (Sarah and Proffitt, 2001; Draganski et al., 2004). An illustrative example is that short-term (3 months) juggling training recruits selective structural change in the same region and has been associated with the processing and storage of complex visual motion (Draganski et al., 2004). The structural differences of handedness in the MT area have also been found in previous studies (Hervé et al., 2006; Steinmetz et al., 2010). In the behavioral study of visual information processing, similar difference between RH and LH/MH (Le Bigot and Grosjean, 2012; Frässle et al., 2016; Smigasiewicz et al., 2017) was also reported. This suggests that the homotopic alterations, as reported in this study, maybe a result of behavioral plasticity.

The orbitofrontal cortex is an important brain area involved in the cognitive process of decision-making, and it is also a brain area with significant individual differences, which received projections from the dorsal nucleus of the medial thalamus, and was considered to represent emotional value and was rewarded in making decisions. Strikingly, the VMHC in the orbitofrontal cortex is significantly different among the three groups. One possibility is that people who are not right-handers have certain personality traits that differ in emotional (Perry et al., 2001; Tranel et al., 2002; Sato and Aoki, 2006) and cognitive processing (Natale et al., 1982), which is consistent with previous hypotheses. Another possibility is that the BOLD signal and the signal-to-noise ratio in the orbitofrontal cortex are poor, so they might not be accurate. We found a significant negative correlation in the orbitofrontal cortex between handedness score and VMHC, suggesting that the closer the handedness score to the extreme of the LH, the stronger the interhemispheric homotopy in the orbitofrontal cortex.

The VMHC reflects interhemispheric information exchange, and its structural basis is mainly based on the corpus callosum, but not limited to. As a bridge connecting the left and right hemispheres of the brain, the corpus callosum is directly involved in the hemispheric transmission of cognitive and sensory information (Hanajima et al., 2010); however, the interhemispheric structural connection is not limited to the corpus callosum, but also extra-callosal pathways, which play an important role in regulating the functional homotopy between the primary sensorimotor and the visual areas (Roland et al., 2017). Clinical studies on agenesis (J Michael et al., 2011), deletion (split-brain) (Johnston et al., 2008) and resection (incision of corpus callosum) (Pizoli et al., 2011) of corpus callosum indicate that this remarkable homotopy depended largely on the integrity of corpus callosum in combination (higher-order) cortex. In addition, multiple extra-callosal pathways might be more likely to be seen in the primary cortex. In brain areas with different VMHC of this study, the orbitofrontal cortex, and the lateral temporal-occipital junction region, as advanced association cortexes, showed more connections in LH/MH than RH. Widespread reports have found that the corpus callosum volume of the LH/MH was larger than that of the RH in the measurement of the difference of the corpus callosum (Witelson, 1985; Habib et al., 1991; Tuncer et al., 2005; Josse et al., 2008). This is consistent with this result that the interhemispheric functional connectivity of LH/MH was stronger than that of RH in these two regions. In electrophysiological studies of rodents and primates, increased neuroelectric activity in the same area was also found (Nardone et al., 2013). As the motor area was connected by the extra-callosal pathways to complete the interaction between the left and right hemispheres, it could present multiple possibilities. As we have seen, the information connectivity of LH in the primary hand motor region was enhanced than that of MH/RH. This was consistent with the result of correlation analysis that the closer to the extreme of the LH, the stronger the connection between the left and right hemispheres.

The gray matter of LH and RH was more rightward shift than that of MH in the medical prefrontal cortex, LH was more leftward shift in STG and SFG compared with MH/RH. The left STG is an important language network node, involving the Wernicke's area and the primary auditory cortex. Although the language network itself has distinct lateralization, there is still little research on the relationship between handedness and the lateralization of the language network. Some previous reports indicated that LH is associated with language ability and language-related diseases, such as stuttering (Chang et al., 2008), epilepsy (Bolin, 1953), and autism (Whitehouse and Bishop, 2008). Some researchers pointed out that in the language of hemispheric dominance, the RH had a typical left hemisphere preference, while the LH had an atypical (right advantage or mixed) language advantage (Knecht et al., 2000; Szaflarski et al., 2002), when forcing to convert a left-hander into a right-hander, the signal transmission was affected, which is prone to stuttering (Kushner, 2012), learning disorder, and deficit disorder with hyperactivity (Goez and Zelnik, 2008). We also found that the LH showed a decrease in rightward on the STG compared with RH, which also suggested that LH could be related to the enhancement of language lateralization in the right superior temporal gyrus. The medial prefrontal cortex and anterior cingulate participate in the expression and evaluation of negative emotions (Amit et al., 2011), and also store long-term regressive memory (Milad and Quirk, 2002). The SFG is involved in self-awareness and motor coordination of the sensory system (Goldberg et al., 2006). Furthermore, when treating epilepsy clinically with electrical stimulation, the patients would laugh when stimulated at the upper frontal gyrus (Fried et al., 1998). We found the differences of AI in the medial prefrontal cortex and the SFG, which indicated that different handedness had different or the same but different degrees of hemispheric dominance in emotional expression and self-awareness.

As for differences in lateralization of handedness, we found that different handedness could have different advantages of brain lateralization in different functions. The atypical lateralization of the non-RH and the typical representation of the RH were not simply mirror-reversed (Michałowski and Króliczak, 2015), even though the dominant direction of hemispheres with different handedness was the same, and the degree of dependence was different. As a study mentioned, RH had an advantage over non-RH in the right brain in facial expression (Bourne, 2008). Bryden et al. have proposed a causal complementary model, for example, the greater the dominant effect of the left hemisphere on language, the greater the dominant effect of the right hemisphere on non-language, and the dominance of the left hemisphere was negatively correlated with the dominant score of the right hemisphere (Bryden et al., 1983). Another study by Bryden (1990) showed that the asymmetry of the frontal lobe generating words was significantly negatively correlated with the temporal lobe asymmetry of face processing and the parietal lobe asymmetry of visual-spatial processing, indicating that there was a complementary relationship between them. This model seemed to explain these results well, for example, in the medical prefrontal cortex, which relied on emotional expression in the left hemisphere, slanting on the right side of gray matter in the LH was higher than that in the MH, while LH slanting on the left hemisphere was higher compared with MH in the SFG of self-consciousness.

We assumed that the emotional expression of LH was lower dependent on the left hemisphere than that of MH and that LH was more dependent on the left hemisphere in the processing of self-consciousness with left hemisphere advantage. The results of the previous study (Willems et al., 2014) described that LH showed increased rates of reductions or reversals of lateralized brain functions, compared with RH. In these results, we did not find a significant dependence reduction in LH than RH in the dominant hemisphere. Some researchers have proposed a single two-allele gene model, in which one allele encoded left-brain dominance and RH, while the other allele did not specify asymmetry, made the direction of handedness and language advantage change at will (Milne and Milne, 1948; Annett, 2013). Perhaps, it was the undirected allele that made hemispheric dominance and handedness irregular in these results. This was generally consistent with our results of the correlation. We found a significantly positive correlation between handedness score and AI, widely distributed in the cerebral cortex, suggesting that the LH was more dependent on the left hemisphere, while the RH was more dependent on the right hemisphere. This complementary functional advantage of the cerebral hemispheres could also be a good indication of the allocation of resources in the cerebral hemispheres, as mentioned earlier in the corpus callosum and the extra-callosal pathways, showing the hierarchical classification of functional differences in cooperation between the cerebral hemispheres, which to promote the efficient functioning of the brain. We found brain areas with significant differences in AI, the medical prefrontal cortex, SFG, and STG. Meanwhile, the lateral temporal-occipital cortices, orbitofrontal cortex, and primary hand motor region (including precentral gyrus, middle frontal gyrus, and SFG) were also found in VMHC analysis. The medical prefrontal cortex, SFG, the orbitofrontal cortex, and the primary hand motor region were the same as the subrange of the frontal lobe. The STG and lateral temporal-occipital cortices belong to the temporal cortex. In general, in this study, the brain areas with significant differences in AI and VMHC were consistent, and the differences in brain functions were reflected in the structure. Meanwhile, they were generally consistent with the results of the correlation analysis.

This study has the following limitations: we have a limited sample size, so the robust results need to be further verified in the analysis of large samples. VMHC and AI analyses are based on the assumption of bilateral hemispheric mirror symmetry, so this treatment may mask the original structure.

In this study, white matter and cerebrospinal fluid signals were removed during preprocessing. In recent years, increasing evidence suggests that white matter signals may also contain useful neural information and support large-scale functional architecture, which may contribute to the large-scale organized architecture of gray matter activity. However, it is still unclear how and to what extent the signals in the white matter reflect behavioral domains including handedness. This important issue may require further research in the future.

## Conclusion

LH/MH showed stronger functional homotopy in the transmodal association regions that depend on the integrity of the corpus callosum, but more variable in primary sensorimotor cortices. We located the specific regions for LH/MH from the perspective of structural and functional specification, suggesting the plasticity of hand movement and different patterns of emotional processing. Besides, the correlation analysis showed that the closer the handedness score to the extreme of the LH, the stronger the interhemispheric functional connectivity, as well as more leftward gray matter. Furthermore, the group differences in VMHC largely align with that in AI.

## Data Availability Statement

The datasets for this article are not publicly available because participants from the present study were assured raw data would remain confidential and would not be shared. Requests to access the datasets should be directed to corresponding author Lei Gao, ncu6096@126.com.

## Ethics Statement

The studies involving human participants were reviewed and approved by The Medical Ethics Committee of Zhongnan Hospital of Wuhan University. The patients/participants provided their written informed consent to participate in this study.

## Author Contributions

HW collected the R-fMRI data of the subjects in the MR scanning. HZ and HW processed the fMRI images, analyzed the data, and drafted the manuscript. HX and LG designed this work and revised the manuscript. YG revised the language expression and grammar. All authors contributed to the article and approved the submitted version.

## Conflict of Interest

The authors declare that the research was conducted in the absence of any commercial or financial relationships that could be construed as a potential conflict of interest.

## Publisher's Note

All claims expressed in this article are solely those of the authors and do not necessarily represent those of their affiliated organizations, or those of the publisher, the editors and the reviewers. Any product that may be evaluated in this article, or claim that may be made by its manufacturer, is not guaranteed or endorsed by the publisher.
